# The effect of shared decision-making on recovery from non-chronic aspecific low back pain in primary care; a post-hoc analysis from the patient, physician and observer perspectives

**DOI:** 10.1186/s12875-022-01624-y

**Published:** 2022-02-02

**Authors:** Ariëtte R. J. Sanders, Niek J. de Wit, Nicolaas P. A. Zuithoff, Sandra van Dulmen

**Affiliations:** 1 Julius centre for health sciences and primary care, University medical centre Utrecht, P.O. box 85500, 3508 GA Utrecht, the Netherlands; 2 Nivel (Netherlands institute for health services research), P.O. Box 1568, 3500 BN Utrecht, the Netherlands; 3 Department of primary and community care, Radboud university edical center, Radboud institute for health sciences, Nijmegen, The Netherlands

**Keywords:** Shared decision-making, Low back pain, Patient participation, Recovery

## Abstract

**Background:**

Although shared decision-making (SDM) is increasingly accepted in healthcare and has demonstrated merits for several psychological outcomes, the effect on recovery from somatic conditions is still subject to debate. The objective of this study is to measure the effect of SDM on recovery from non-chronic aspecific low back pain (LBP).

**Methods:**

This study is a post-hoc analysis of data from a cluster-randomised trial that evaluated the effectiveness of SDM on recovery in patients with non-chronic aspecific LBP. In this analysis, we re-evaluate the impact of SDM from three perspectives: that of external observers, participating GPs and participating patients. Recovery was measured with the Visual Analogue Scale (VAS) for pain and with the Roland Morris Disability questionnaire (RMD) and defined as a VAS < 30 and an RMD < 4. Logistic regression was used to analyse the effect of SDM on recovery at 6 and 26 weeks.

**Results:**

At 26 weeks, 105 (74%) of all 176 included patients had recovered. No significant effect of SDM on recovery at 6 or 26 weeks after the consultation was found when considering SDM from an observer perspective or a patient perspective. From a GP perspective SDM had a significant effect on recovery, but at 26 weeks only, and with the lowest probability of recovery observed at a medium level of GP-perceived SDM.

**Conclusions:**

We found no evidence that SDM as perceived by the patient or by external observation improves recovery from non-chronic aspecific low back pain. The long-term recovery may be better for patients in whom the GP perceives SDM during their consultations. Further research should highlight the hierarchy and the relation between the perspectives, which is needed to come to an integral effect evaluation of SDM.

**Trial registration:**

The Netherlands National Trial Register (NTR) number: NTR1960.

**Supplementary Information:**

The online version contains supplementary material available at 10.1186/s12875-022-01624-y.

## Background

Aspecific low back pain (LBP), i.e. back pain without a known specific somatic origin, is among the top 10 most frequently presented complaints in primary care [1]. It subsides within 2 weeks in the majority of patients but can become chronic (> 3 months) or frequently recurring (≥3 episodes a year) [1]. Worldwide, it is one of the leading causes of disability, with a societal burden primarily incurred through costs related to losses in productivity (93% of total costs) [2, 3].

Professional guidelines for LBP commonly recommend assessing patients’ perceptions and informing patients properly about the expected favourable course of the complaints [1, 4]. In addition, it has been suggested that patient involvement in the medical decision-making process has a positive effect on the course of illnesses like LBP [5].

A process in which the professional and the patient share their perspectives and jointly decide on a treatment plan, is shared decision-making (SDM) [6]. Elwyn identified four key steps in SDM: (1) the professional informs the patient that a decision needs to be made and that the patient’s opinion is important; (2) the professional explains the options and their pros and cons; (3) the professional and the patient discuss the patient’s preferences, and the professional supports the patient in their deliberation; (4) the professional and patient discuss the patient’s wish to make the decision, they make or defer the decision and discuss follow-up [6]. Although the concept is increasingly accepted in healthcare, the implementation of SDM in clinical practice varies significantly, depending on the perspective of patients, providers or external observers [7].

Although the benefit of SDM has been demonstrated for several psychological outcomes, such as the patient’s emotional status, the effect on recovery from somatic conditions is still subject to debate [8]. In SDM, patients’ concerns are explored, which might increase their feelings of being taken seriously and might improve trust in the professional [9]. Moreover, in SDM patients’ preferences and outcome expectations are taken into account when jointly deciding on the treatment plan. In a symptom-based illness like LBP, recovery seems associated with patients’ outcome expectations, the attitude of the professional and the relationship with the professional [10–13]. Therefore theoretically, if the outcome expectations of the patent are reinforced in the context of a mutually agreed therapeutic plan within a patient-doctor relationship in which the patient feels supported, one would expect LBP complaints to subside more quickly compared to traditional care which is professional driven and therapy focussed [14–16].

We previously reported the results of a randomised controlled trial (RCT) in general practice examining the effects of training GPs in SDM in patients suffering from non-chronic LBP, with SDM measured from the observer perspective only [9, 17, 18]. We could not detect a significant benefit from SDM on patient recovery, as objectified with the Roland Morris Disability scale or Visual Analogue Pain scale [19, 20]. This could potentially be explained either by a lack of contrast between the intervention and control groups in the level of SDM in practice because of inadequate application of SDM in the intervention group, or by differences between patients and GPs in perceptions of SDM during the consultations in the two study arms. To assess this, further detailing is needed of the association between recovery independently of the allocation and the level of SDM during the consultations from a broader view than just the observer perspective (‘treatment fidelity’).

We therefore performed a post-hoc analysis of the RCT data and re-evaluated the effectiveness of SDM, as perceived from three perspectives: that of external observers, participating GPs and participating patients.

## Methods

### Design

This is a post-hoc analysis of data from a clustered randomised trial that evaluated the effectiveness of SDM among patients with non-chronic LBP. Details of the design, intervention and overall outcome are described elsewhere [18, 21].

**Fig. 1:**
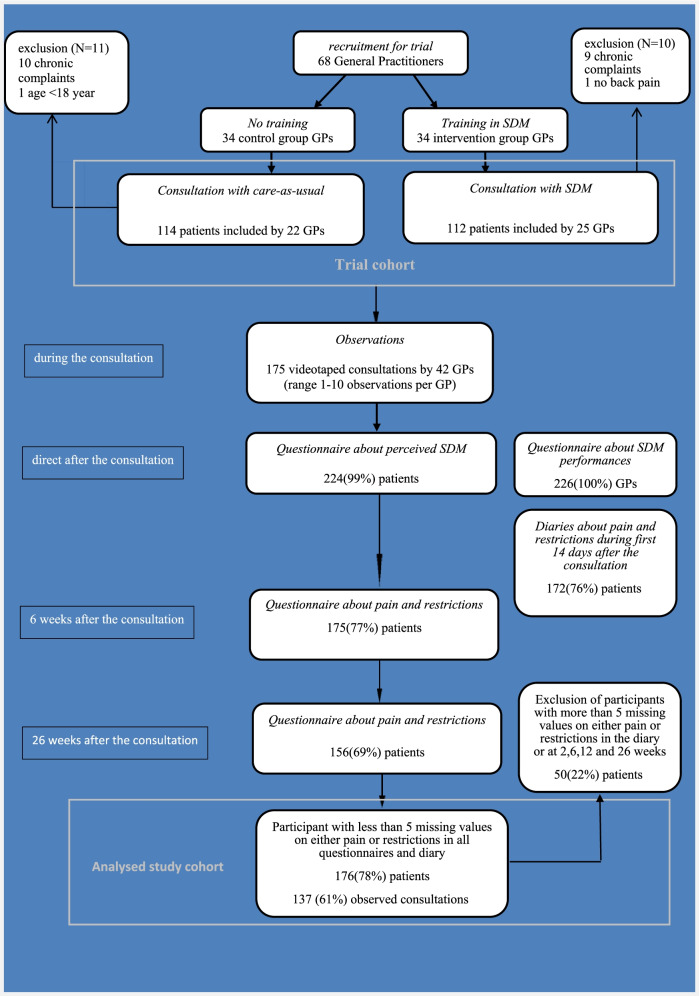
Flowchart

For the current analysis, we merged all patients from the intervention and control groups into a prospective cohort, including follow-up measurements until 26 weeks after the initial consultation. We excluded patients who missed more than 5 outcomes for either restrictions or pain on all 19 time points (questionnaires at baseline, 2, 6, 12 and 26 weeks, and a diary for the first 14 days after the consultation) (Fig. 1).

### Participants

Adult patients (aged between 18 and 65 years) who contacted their GP because of a new episode of non-chronic LBP, as defined by the guideline of the Dutch College of General Practice, were invited to participate in the trial between August 2009 and May 2011 [4]. Exclusion criteria were: duration of LBP longer than 3 months, recurring backache within 3 months of the primary episode, pregnancy and insufficient mastery of the Dutch language.

Participating GPs were part of the primary care network around Utrecht (the Netherlands) affiliated with the university. They completed a questionnaire about baseline information after they were recruited for the trial [18]. Intervention GPs were trained in SDM [21]. Control GPs delivered care-as-usual. During the study period, 68 GPs included 226 patients with non-chronic LBP (Fig. 1) [18].

### Data collection

GPs were asked to reflect on their SDM performance directly after each individual consultation. Patients gave permission for the video-recording of their consultation and were asked to complete questionnaires before and after the consultation with the GP [18]. From all 226 patients in the trial, we had video-recordings of 86 consultations conducted by 23 GPs in the intervention group and 89 consultations conducted by 19 control-group GPs (Fig. 1) [21].

### Measurements and instruments

#### LBP-related outcomes

Pain severity was quantified by the validated Visual Analogue Scale (VAS), referring to patients’ self-reported level of pain during the past week, ranging from 0 to 10 [22].

Perceived functional disability was assessed by the Dutch version of the Roland-Morris Disability Questionnaire (RMD). This validated questionnaire contains 24 questions about restrictions in daily activities during the past day, which patients had to tick when applicable. Scores are summed as the number of positive answers [23].

The primary outcome was recovery at 26 weeks, which was defined by a VAS of 30 mm (scale 0–00) or less combined with a maximum of three disabilities on the RMD questionnaire (scale 0–24) at 26 weeks [20]. As a secondary outcome, we assessed recovery at 6 weeks, which was defined by a VAS of 30 mm or less (scale 0–100 mm) combined with a maximum of three disabilities on the RMD (scale 0–24) at 2 weeks after the consultation [20].

#### Recorded SDM

SDM was assessed from the three different perspectives.

The observer-reported SDM was assessed using the OPTION scale, a validated observation instrument for measuring the extent to which a healthcare provider involves a patient in SDM [24]. The scale distinguishes 12 process elements of SDM (ranging from 0 to 4), and these scores are summed to obtain one overall score. A score of 0 corresponds to ‘no behaviour observed’, and a score of 4 indicates that the behaviour is exhibited to a high degree. The sum scores of the 12 process elements are transformed into a scale from 0 to 100 [21].

Patient-reported SDM was evaluated by a single question on a 4-point scale directly after the consultation: “How much were you involved in decision-making” (‘not at all’, ‘not really’, ‘on the whole, yes’ and ‘yes’). The answers were scored 1 to 4 where ‘yes’ =4 and transformed into a scale from 0 to 100. This non-validated but easily applicable measure requires a patient to provide a global assessment rather than a reflection on the separate process steps. Single-item, generic patient-reported measurements are simple and easy to understand, and have demonstrated comparable validity and reliability to multi-items scales in other fields like quality-of-life measurements [25].

To measure GP-reported SDM, we developed a GP questionnaire, transforming the description of each previously mentioned process element of the OPTION scale into one question about the GP’s self-reflection on the level of performance of the corresponding SDM process element. We left out item three (inquiry into the preferred information format), because it might reveal the intervention to the control GPs. In the OPTION, this element is hardly ever scored and has hardly any influence on the overall scale [26]. Scores were on a 4-point scale ranging from ‘not at all’, ‘not really’, ‘on the whole yes’ to ‘yes’. All questions were formulated by AS, checked for content by two research students (DA and ME) and tested on two non-participating GP colleagues. The sum score of the 11 process elements was transformed into the qualifying answers: ‘not at all’ =1, ‘not really’ =2, ‘on the whole yes’ =3, and ‘yes’ =4. The different process items were summed to give one overall score and transformed into a scale from 0 to 100.

### Statistical analysis

Baseline characteristics (i.e. just before, during or directly after the consultation) were reported as means and standard deviations or N and percentages, as applicable. Recovery at 6 and 26 weeks was analysed using logistic regression analysis for each of the three SDM assessments separately. In a first step, we estimated the effect of SDM as reported by the patients and GPs or SDM as scored by independent observers without any adjustment. In a second step, we included the patient’s age, sex, educational level, absenteeism and baseline measurements of both the VAS and RMD as potential confounders [27].

The assumption of linearity of continuous variables, including the SDM scores, was assessed with restrictive cubic splines and tested with likelihood ratio tests (Supplementary file 1) [28].

Prior to preforming the analysis, we noted substantial missing values for multiple variables, including missing values for the VAS and the RMD at different time points during follow-up, including 6 and 26 weeks, where these scores are used to determine the outcomes for this study. Most of these missing values were due to patients not returning the diary used to assess VAS and RMD during the first 14 days of follow-up.

Fifty patients were excluded because they were missing at least five of the 18 outcomes for either VAS or RMD. In most of the cases this was due to diary measurements that were incomplete or not returned (Fig. 1). Baseline characteristics and recovery rates of all 50 excluded patients and their GPs are given in supplementary file 2, supplementary Table 1. For the remaining 176 patients, we used multiple imputation techniques. We imputed missing values for the VAS and RMD measurements over time, SDM as reported by the patient and the GP and the SDM scored by independent observers, the patient’s sex, age, absenteeism from work at baseline and scores for the Illness Perception Questionnaire (IPQ). Patient perceptions of low back pain can be confounders and were measured during the consultations as described in the original trial [18]. For patients with missing values for VAS or RMD at 6 or 26 weeks, outcomes were determined based on the imputed scores. The number of imputations was based on the percentage of patients with one or more missing values. We imputed the data 67 times and performed all analyses on each imputed dataset. Results were pooled according to Rubin’s rule. (18) Results were reported as odds ratios with 95% CIs and corresponding *p*-values [29].

Spearman’s correlations of the non-imputed data were calculated between observer-reported SDM, patient-reported SDM and GP-reported SDM.

## Results

### Baseline characteristics

GP and patient characteristics, patient recovery rates and the numbers of missing data are provided in Table 1 for the participants in this post-hoc analysis. Participating patients and GPs in the constructed database did not differ from the original trial cohort in any of the variables presented in Table 1. The mean level of patient-rated pain at baseline was 48.90 (sd 15.70) on a scale from 0 to 100 and they perceived a disability in 10.03 (sd 5.75) of the 24 items on average. At 6 weeks after consultation 101 (66%) patients were recovered, and at 26 weeks 105 (74%) patients were recovered (Table 1). The mean level of observer-rated SDM was 30.76 (sd 36.73), which is less than half the value recommended for best practice based on the maximum score of 100. The patient-perceived level of involvement in decision-making was 78.03 (sd 36.73), and GPs scored their SDM performance on average as 53.46 (sd 20.23), all on a scale from 0 to 100 (Table 1). Almost one quarter (24%) of all patients experienced no involvement at all (Supplementary file 2, supplementary Table 2). GPs indicated that in 22% of the cases they did not involve patients in at least one of the 11 steps of decision-making (Supplementary file 2, supplementary Table 2). Spearman’s correlations between the three SDM measurement perspectives were low (< 0.150) except for a moderate, significant correlation of 0.418 between observer-reported SDM and GP-reported SDM (Supplementary file 2, supplementary Table 3).

**Table 1:** Baseline and recovery characteristics of all 176 patients, GP characteristics and the level of SDM

	number or mean	percentage or standard deviation (sd)	number of missing values (percentage)
***patients characteristics***			
male	80	46.2%	3 (0.02%)
mean age	46.77	13.16 sd	0
educational level			7 (0.04%)
primary school educational attainment only	25	14.8%	
at least secondary school educational completion	84	49.7%	
at least college, university completion	60	35.5%	
aAbsenteeism from work (yes/no)	70	30.3%	24 (14%)
intervention group	91	51.7%	0
***disease characteristics at baseline***			
pain severity (VAS; 0–100)	48.90	15.70 sd	27 (15%)
functional disability score (RMD; 0–24)	10.03	5.75 sd	5 (0.03%)
***GP characteristics***			
male	27	57%	0
mean age	51.38	7.029 sd	0
educator	33	70%	0
years’ experience as GP	18.02	7.820 sd	0
mean number of patients included	5.06	3.03 sd	0
***SDM***			
observer-reported ESDM (OPTION scale; 0–100)	30.76	10.82 sd	41 (23%)
pPatient-reported SDM (scale 0–100)	78.03	36.73 sd	3 (0.02%)
GP-reported SDM (scale 0–100)	53.46	20.23 sd	8 (0.05%)
***recovery***^**a**^			
recovered at 6 weeks	101	66.45%	24 (14%)
recovered at 26 weeks^b^	105	73.94%	34 (19%)

^a^recovery defined by a VAS-score < 30 mm and a RMD ≤ 3 restrictions

^b^primary outcome

### The effect of SDM on recovery at 26 weeks

In the unadjusted analysis the observer-reported SDM process steps measured using the OPTION scale were not significantly associated with recovery at 26 weeks after the consultation (OR 1.026 (95% CI: 0.986–1.068, *p*-value = 0.206). After adjustment for confounders, the OR was 1.033 (95% CI: 0.987–1.080, p-value = 0.156).

Patient-reported SDM was also not significantly associated with recovery when unadjusted (OR 1.002 (95% CI: 0.992–1.012, *p*-value = 0.745) and after adjustment (OR 0.998 (95% CI: 0.987–1.009, p-value = 0.723). GP-reported SDM had a non-linear association with recovery. To solve the problem of non-linearity, splines were introduced in the analysis of GP-scores with cut-off points below 40 (indicating almost no SDM according to the GP) or above 70 (indicating high levels of SDM according to the GP). Scores below 40 showed an odds ratio of 0.965 (95% CI: 0924–1.008, *p*-value = 0.111); medium-rated SDM (scores from 40 to 70) showed an odds ratio of 1.021 (95% CI 0.995–1.047, p-value = 0.110); and a high level of SDM (scores above 70) showed an odds ratio of 1.069 (95% CI: 1.010–1.132, p-value = 0.021). After adjustment ORs were 0.985 (95% CI: 0.9140–1.008, *p*-value = 0.960) for scores below 40, 1.022 (95% CI: 0.993–1.051, p-value = 0.145) for scores between 40 and 70 and 1.076 (95% CI: 1.007–1.150, *p*-value = 0.031) for scores above 70 (Table 2).

**Table 2:** Unadjusted and adjusted logistic regression of recovery as a function of the prognostic variables after multiple imputation. Unadjusted- and adjusted logistic regression of prognostic variables on recovery after multiple imputation

	recovered at ***26*** weeks^**a**^						recovered at ***6*** weeks^**a**^					
	unadjusted logic regression			adjusted logistic regression			unadjusted logic regression			adjusted logistic regression		
	odds ratio	confidence interval	*p*-value	odds ratio	confidence interval	*p*-value	odds ratio	confidence interval	*p*-value	odds ratio	confidence interval	*p*-value
***SDM***												
observer-reported SDM (measured by OPTION)	1.026	0.986–1.068	0.206	1.033	0.9887–1.080	0.15655	1.007	0.975–1.041	0.663	1.0168	0.97981–1.0557	0.39937
patient-reported SDM	1.002	0.992–1.012	0.745	0.998	0.987–1.009	0.7232	0.997	0.988–1.007	0.587	1.0065	0.995–1.016	0.30123
GP-reported SDM (hardly) no SDM at all (< 40)	0.965	0.924–1.008	0.111	0.98560	0.91407–1.008	0.960102	1.006	0.990–1.023	0.458	1.006	0.988–1.025	0.509487
GP-reported SDM intermediate level SDM (40–70)	1.021	0.995–1.047	0.110	1.022	0.993–1.051	0.145						
GP-reported SDM (relatively) high level SDM (> 70)	1.069	1.010–1.132	0.021*	1.0765	1.007–1.15049	0.031*						
***patient characteristics***												
male	1.230	0.846–1.789	0.278				1.249	0.894–1.743	0.193			
mean age	0.9777	0.950–1.006	0.123				1.012	0.987–1.038	0.339			
educational level secondary school educational completion related to primary school educational attainment only	0.490	0.260–0.924	0.0276*				0.520	0.291–0.930	0.028*			
educational level college, university educational completion related to primary school educational attainment only	1.299	0.782–2.156	0.3126				1.232	0.776–1.055	0.376			
***disease characteristics***												
pain severity (VAS; 0–100)	0.969	0.943–0.994	0.017*				0.956	0.933–0.981	0.001*			
functional disability score (RMD; 0–24)	1.026	0.953–1.104	0.497				0.988	0.926–1.054	0.715			

^a^recovery defined by a Visual Analogue Scale < 30 mm and a Roland Morris Disability questionnaire ≤3 restrictions after the consultation

*p-value< 0.05

### The effect of SDM on recovery at 6 weeks

In the adjusted analysis the observer-reported SDM process steps measured by the OPTION scale showed no effect on recovery at 6 weeks after the consultation, with an odds ratio of 1.016 (95% CI: 0.979–1.055, *p*-value = 0.399). Patient-reported SDM and GP-reported SDM also showed no effect on recovery, with odds ratios of 1.016 (95% CI: 0.995–1.1.016, p-value = 0.301) for patient-reported SDM and 1.006 (95% CI: 0.988–1.025, p-value = 0.509) for GP-reported SDM (Table 2).

In the unadjusted analysis a higher baseline level of pain significantly decreased the likelihood of recovery at 6 and 26 weeks. The impact is modest (< 5% less chance of recovery per 10 mm on the VAS scale from 0 to 100 mm) (Table 2).

## Discussion

In this post-hoc analysis we assessed the impact of SDM (as assessed from the patient, GP and observer perspectives) on long-term and short-term recovery from non-chronic LBP. From any of these three perspectives SDM did not improve recovery from LBP at 6 or 26 weeks, except for the GP-reported level of SDM, which was associated with recovery at 26 weeks.

From a GP’s point of view, there was a non-linear significant effect on recovery. The lowest probability of recovery was observed at a medium level of GP-reported SDM, where each increase in the level of SDM (on the 4-point scale) per single process step of the 11 steps of GP-reported SDM increased the patient’s recovery chance at 26 weeks by 2.3%.

The results of this post-hoc analysis confirm the conclusion from our trial: there is no convincing evidence that SDM improves outcomes in patients with LBP, despite the fact that patients with LBP indicate a need for patient-centred care and active involvement and the fact that recovery from LBP is associated with patents’ and GPs’ recovery expectations [9, 17]. The results of this study further strengthen this conclusion of absence of a detectable effect by considering different angles for the evaluation of SDM: that of patients, of GPs and of external observers. We could not identify an integral SDM effect from these different perspectives. Only one of the six SDM measurements tested was significantly associated with recovery. Moreover a post-hoc analysis increases the risk that this significance may be caused by multiple testing rather than be a true effect. The fact that GP-perceived SDM was found to be associated with favourable long-term recovery may equally be explained by a professional perception that was not shared by patients, and not confirmed in observation [17].

.Since the introduction of SDM in 1982 by the President’s Commission for the Study of Ethical Problems in Medicine and Biomedical and Behavioral Research in its report Making Health Care Decisions, there has been an ongoing debate about the concept and how to measure optimal performances [30–36].] It is worthwhile returning to this original report as while we do not wish to ignore the knowledge that has been accumulated since then, nor do we want to fall into the trap of restricting ourselves to an elaboration of the concepts that currently happen to receive most attention. The original report describes SDM clearly as ‘a process based on mutual respect and partnership, that will usually consist of discussions between professional and patient that bring the knowledge, concerns, and perspective of each to the process of seeking agreement on a course of treatment.’ Clearly, health professional and patient both have responsibility in this process: the health professional creates the opportunity for a dialogue and makes sure the patient understand the medical situation and available courses of action, the patient expresses prevailing concerns, needs and wishes. In addition, the practitioner offers alternative courses of action to allow the patient to make a decision according to his views on well-being [30].

In 2019, after a systemic review of SDM models, Bomhof provided a map of 24 components of SDM elements [31]. Like Joseph-Williams et al., they indicated that exclusive or essential SDM behaviour should be separated from more general, context-related communication skills that serve as facilitators [31, 32]. But these general skills play a crucial role in creating an environment to optimise the exclusive elements [32]. Since SDM is displayed in communicative behaviour and perceived in patients’ minds, one might argue that even exclusive elements actually serve as facilitators to reach the outcome of ‘a shared choice’, defined as a mutually agreed plan of action preferred by the patient but achieved after a respectful dialogue where the knowledge, concerns and perspective of each are shared [30, 33, 34].

The measurement of SDM should therefor at least include the patient and the outcome. Unfortunately, patients’ evaluation of ‘a shared choice’ seems based solely on the level of mutual agreement without incorporating the quality of the information exchange or deliberation [35]. Patient-reported (and provider-reported) measurements of process elements suffer from ceiling effects, possibly due to the halo effect, defined as incorporating the whole encounter, the ongoing relationship with the clinician or clinician attributes [7, 36].

Observer-based coding schemes, requiring raters trained in the evaluation of SDM, usually apply stricter criteria and reveal lower levels of SDM compared to results based on self-report instruments [26]. But external raters cannot accurately determine the level of ‘a shared choice’ since this is predominantly perceived in patient’s mind [35]. Although reflection (‘stop-and-think’) before rating did not mitigate ceiling/halo effects, training patients (or providers) as raters of observed or audio-recorded encounters might increase the performance of self-reported SDM measures [36].

### Methodologic considerations

Several limitations need to be addressed. Illness-related characteristics, like levels of experienced pain or disabilities, patient’s characteristics, like their mood, behaviour or socio-economic status, or even GP characteristics and the interaction between GP and patients might also influence the prognosis of non-chronic LBP. However, adjustment for these variables, which we did in the second step of our analysis, did not change any results [27]. Even in a short time span of 6 weeks we did not find any evidence that SDM might have influenced recovery. But enhanced health outcomes are not the only aim of SDM. Besides ethical considerations, SDM aims to limit practice variation and thus decrease inequality, promote patient autonomy and ensure that treatment decisions reflect patient preferences [6].

A possible explanation of the observation that GP’s reflections on their moderate performance of SDM aligns with a higher chance of developing non-chronic complaints might be that GPs adapt their behaviour to the patient’s characteristics associated with recovery rates [12, 37, 38]. Recently, Arnborg Lund described GPs’ views on treating LBP as an act of dialogue rather than a fragmented experience with different explanations and recommendations [39].).

The OPTION scale used by independent observers of SDM is an externally validated scale. For both patient-reported SDM and GP-reported SDM, easily applicable validated instruments were not available at the time of this study. Even in consultations for a relatively simple complaint like LBP, patients might experience difficulty in recognising involvement in the decision being assessed [40]. Therefore we decided to use the single question measurement for patient SDM assessment because this is easily applicable and simple to understand. However, we do realise that this measurement is not validated. The overall level of observer-reported SDM of the cohort was low (less than 50% of the maximum score), although comparable to other studies [14, 26]. Substantially higher levels of observer-reported SDM behaviour are rarely measured in controlled trials [26]. It is unclear what effect substantial observer-reported SDM would have on recovery [26].

An important methodological limitation is the high number of missing values. Fifty patients were considered lost to follow-up and excluded from the analysis, as the number of missing values was deemed too high. In additional analyses, we detected no clear association between loss to follow-up and SDM measurements or baseline measurements of the VAS or the RMD. For the remaining 176 patients, we used multiple imputation. In line with current recommendations, we based the number of imputations on the percentage of patients with one or more missing values [29]. When evaluating the percentage of patients with missing values, we considered two factors. First, a large proportion of the remaining patients had only a few missing values (Supplementary file, supplementary Table 2). The VAS (over time) was the variable with the highest percentage of missing values. Second, we incorporated all VAS and RMD scores over time, including measurements not used to define the outcome, as consecutive measurements of VAS and RMD scores showed correlations of 0.80. We incorporated these measurements to obtain the best possible imputation model, even though the number of patients with any missing values increased. Nevertheless, a bias due to either loss to follow-up or missing values cannot fully be excluded.

## Conclusion

In a post-hoc-analysis of RCT data on primary-care patients suffering from non-chronic LBP, we found no convincing evidence that SDM improves recovery from LBP, neither in the long term nor in the short term. These results were unaffected by the perspective from which SDM was measured (observer, patient perception or GP reflection). Further research should focus on the consistently high performance of SDM to determine whether SDM influences recovery at all.

## Supplementary Information

**Additional file 1.** Additional information regarding the methods of the analysis.

**Additional file 2. ** Supplementary tables.

## Data Availability

The datasets used and/or analysed during the current study are available from the corresponding author on reasonable request.

## Abbreviations

CI: Confidence interval
IPQ: Illness perception questionnaire
GP: General practitioner
LBP: Low back pain
NTR: Netherlands trial register
RCT: Randomised controlled trial
RMD: Roland Morris disability questionnaire
sd: Standard deviation
SDM: Shared decision-making
VAS: Visual analogue scale for pain
