# Supplementary material for: The effect of shared decision-making on recovery from non-chronic aspecific low back pain in primary care; a post-hoc analysis from the patient, physician and observer perspectives
Source: BMC Prim Care. 2022 Feb 2;23:22. doi: 10.1186/s12875-022-01624-y (PMC8809011; doi:10.1186/s12875-022-01624-y)
Supplement: Supplementary file 1 — Additional file 1. Additional information regarding the methods of the analysis. [file 12875_2022_1624_MOESM1_ESM.docx]

Supplementary file 1 ‘Additional information regarding the methods of the analysis’

**Restrictive cubic splines in regression**

Most often used regression methods (i.e. linear, logistic, Cox regression) rely on an assumption of linearity for continuous explanatory variables such as age. For age in a logistic model, this may be seen as an assumption that the odds ratio for an increase from 30 to 40 years is the same compared to an increase from 60 to 70 years of age. This, however may not always be the case: the association may for example follow a parabolic form (to be modelled with a squared term) or any other form. This is sometimes referred to as the functional form of the association. Key problem is that a functional form is often unknown prior to a statistical analysis.

In this study, two of the three SDM assessments as well as age were included in the analysis as continuous explanatory variables. As a violation of this assumption may lead to biased results, we tested the assumption (for each variable and each outcome separately) with restrictive cubic splines.

In this document, we provide a somewhat simplified explanation of splines as well as additional information on how these were used in the analysis. Even though we made every effort to provide a valid, yet easy to understand explanation, this document should by no means be considered as a full explanation on the use of restrictive cubic splines. We refer interested readers to Harrell (2015, chapter 2) for more detailed explanation as well as additional references. (Harrell, 2015)

The basic method of a spline involves dividing the distribution of a continuous variable into parts, i.e. the lowest value to the 20^th^ percentile, 20^th^ to 40^th^ percentile, etc. The cut-points are referred to as knots. It is important to realize that this is not a form of categorization (i.e. the lowest value to the 20^th^ percentile becomes category 1), the values of individual patients within a certain part is left continuous. Instead of estimating a single OR, separate ORs are estimated for each part the distribution was divided in. Hence in the example above, 5 separate ORs may be estimated, introducing more flexibility in the assumption of linearity, whatever the functional form of the association may be.

This somewhat simplified example of a spline has several downsides: within a regression model the number of coefficients (i.e. log odds ratio’s) that need to be estimated may become large and stability of the statistical model may be problematic. An more advanced approach is the restrictive cubic spline (also called natural splines), which involves the inclusion of so called higher order (e.g. Age^3^). This method provides similar advantages in terms of flexibility with more stable results while estimating less regression coefficients.

**Application**

During the first phase of the analysis, we tested restrictive cubic spline (RCS) and compared these with a likelihood ratio test. First tests were performed in a univariate model, any non-linear effect was subsequently evaluated in a multivariate model. When applying splines, a decision needs to be made on the number of knots to be used and where these knots need to be placed. Initially, we relied on the default settings of the software (SAS v9.4) and evaluated increasing number of knots. Additionally, we evaluated the location of knots over the multiple imputed dataset, as the above described method allows for different knot locations in different imputed datasets. A non-linear effect was only observed for GP-reported SDM. Although some differences in the knot locations for this variable were observed, in general, knots located at values of about 40, 55 and 70 seemed generally acceptable. These knots were subsequently used in the analysis of all imputed data to allow for pooling of the results and estimation of pooled odds ratio’s with corresponding 95% confidence intervals and p-values.
