# Supplementary material for: The effect of shared decision-making on recovery from non-chronic aspecific low back pain in primary care; a post-hoc analysis from the patient, physician and observer perspectives
Source: BMC Prim Care. 2022 Feb 2;23:22. doi: 10.1186/s12875-022-01624-y (PMC8809011; doi:10.1186/s12875-022-01624-y)
Supplement: Supplementary file 2 — Additional file 2. Supplementary tables. [file 12875_2022_1624_MOESM2_ESM.docx]

Supplementary file 2 ‘supplementary tables’

Supplementary table 1. Baseline characteristics and recovery rates of all 50 excluded patients and their GPs.

|  | number or mean | percentage or standard deviation (sd) | number of missing values |
| --- | --- | --- | --- |
| ***patients characteristics*** | | | |
| male | 27 | 54 % | 1 |
| mean age | 38.14 | 14.07 sd | 0 |
| educational level |  |  | 1 |
| primary school educational attainment only | 9 | 18 % |  |
| at least secondary school educational completion | 25 | 50 % |  |
| at least college, university completion | 15 | 30 % |  |
| absenteeism from work (yes/no) | 28 | 56% | 5 |
| intervention group | 21 | 42 % | 0 |
| ***disease characteristics at baseline*** | | | |
| pain severity (VAS; 0-100) | 43.923 | 17.93 sd | 7 |
| functional disability score (RMD; 0-24) | 9.960 | 4.907 | 0 |
| ***disease characteristics at 6 weeks*** | | | |
| pain severity (VAS; 0-100) | 13.890 | 15.33 sd | 43 |
| functional disability score (RMD; 0-24) | 4.143 | 5.76 sd | 43 |
| ***disease characteristics at 26 weeks*** | | | |
| pain severity (VAS; 0-100) | 19.619 | 26.70 sd | 43 |
| functional disability score (RMD; 0-24) | 2.833 | 3.37 sd | 44 |
| ***SDM*** | | | |
| observer-reported (OPTION scale; 0-100) | 29.955 | 11.35 sd | 13 |
| patient-reported SDM (scale 0-100) | 75.694 | 35.56 sd | 2 |
| GP-reported SDM (scale 0-100) | 56.000 | 20.73 sd | 0 |
| ***recovery†*** | | | |
| recovered at 6 weeks | 3 | 42.9 % | 43 |
| recovered at 26 weeks# | 4 | 57.1 % | 43 |
| ***GP characteristics*** | | | |
| male | 16 | 59.3 % | 0 |
| mean age | 53.296 | 7.05 sd | 0 |
| educator | 21 | 77.8 % | 0 |
| years’ experience as GP | 20.667 | 7.92 sd | 0 |

† recovery defined by a VAS-score <30 mm and a RMD ≤ 3 restrictions after the consultation; #Primary outcome

Supplementary table 2. Levels of SDM from all three perspectives of the analysed cohort of 176 cases. Absolute numbers (percentage)

|  | not observed | minimal attempt | minimum level | good level | very high level | not at all | not really | on the whole yes | yes | number of missing values |
| --- | --- | --- | --- | --- | --- | --- | --- | --- | --- | --- |
| ***SDM*** | | | | | | | | | | |
| ***observer-reported SDM*** | | | | | | | | | | |
| GP draws attention to a decision-making stage | 44  (25%) | 78  (44%) | 9  (5%) | 2  (1%) | 4  (2%) |  |  |  |  | 39  (22%) |
| equipoise | 89  (51%) | 14  (8%) | 24  (14%) | 2  (1%) | 8  (5%) |  |  |  |  | 39  (22%) |
| information format | 83  (47%) | 48  (27%) | 2  (1%) | 3  (2%) | 1  (0%) |  |  |  |  | 39  (22%) |
| lists options | 20  (11%) | 0  (0%) | 32  (18%) | 27  (15%) | 58  (33%) |  |  |  |  | 39  (22%) |
| explanation of pros and cons of options | 31  (18%) | 47  (27%) | 52  (30%) | 5  (3%) | 2  (1%) |  |  |  |  | 39  (22%) |
| exploration of the patient’s expectations | 5  (3%) | 47  (27%) | 79  (45%) | 5  (3%) | 1  (0%) |  |  |  |  | 39  (22%) |
| exploration of the patient’s concerns | 129 (73%) | 7  (4%) | 1  (0%) | 0  (0%) | 0  (0%) |  |  |  |  | 39  (22%) |
| check of patient’s understanding | 13  (7%) | 105  (60%) | 19  (11%) | 0  (0%) | 0  (0%) |  |  |  |  | 39  (22%) |
| offering opportunities to ask questions | 20  (11%) | 66  (38%) | 50  (28%) | 0  (0%) | 0  (0%) |  |  |  |  | 40  (23%) |
| elicitation of patient’s preferred level of involvement | 25  (14%) | 42  (24%) | 48  (27%) | 12  (7%) | 10  (6%) |  |  |  |  | 39  (22%) |
| indication of a decision-making stage | 81  (46%) | 54  (31%) | 1  (0%) | 0  (0%) | 0  (0%) |  |  |  |  | 40  (23%) |
| indication of the need to review the decision | 32  (18%) | 10  (6%) | 7  (4%) | 1  (0%) | 87  (49%) |  |  |  |  | 39  (22%) |
| ***patient-reported SDM*** | | | | | | | | | | |
|  |  |  |  |  |  | 42  (24%) | 11  (6%) | 40  (23%) | 76  (43%) | 7  (4%) |
| ***GP-reported SDM*** | | | | | | | | | | |
| GP draws attention to a decision-making stage |  |  |  |  |  | 31  (18%) | 56  (32%) | 41  (23%) | 44  (25%) | 4  (2%) |
| equipoise |  |  |  |  |  | 42  (24%) | 46  (26%) | 43  (24%) | 43  (24%) | 2  (1%) |
| lists options |  |  |  |  |  | 10 (6%) | 37  (21%) | 39  (21%) | 87  (50%) | 3  (2%) |
| explanation of pros and cons of options |  |  |  |  |  | 47  (27%) | 36  (20%) | 49  (28%) | 42  (24%) | 2  (1%) |
| exploration of the patient’s expectations |  |  |  |  |  | 24  (14%) | 38  (22%) | 65  (37%) | 46  (26%) | 3  (2%) |
| exploration of the patient’s concerns |  |  |  |  |  | 19  (11%) | 55  (31%) | 56  (32%) | 43  (24%) | 1  (0%) |
| check of patient’s understanding |  |  |  |  |  | 16 (9%) | 35  (20%) | 58 (33%) | 63 (36%) | 4  (2%) |
| offering opportunities to ask questions |  |  |  |  |  | 1 (0%) | 27  (15%) | 59 (34%) | 86 (49%) | 3  (2%) |
| elicitation of patient’s preferred level of involvement |  |  |  |  |  | 53 (30%) | 54  (31%) | 44 (25%) | 23 (13%) | 2  (1%) |
| indication of a decision-making stage |  |  |  |  |  | 96 (55%) | 36  (20%) | 22 (13%) | 19 (11%) | 3  (2%) |
| indication of the need to review the decision |  |  |  |  |  | 79 (45%) | 35  (20%) | 38 (22%) | 21 (12%) | 3  (2%) |
|  |  |  |  |  |  | 418  (22%) | 399  (21%) | 514  (27%) | 517  (28%) | 30  (2%) |

Supplementary table 3. Spearman’s correlations between observer-reported SDM, patient-reported SDM and GP-reported SDM all scaled 0-100 of the *non-imputed* post-hoc analysis cohort

|  |  | ***observer-reported SDM*** | ***patient-reported SDM*** | ***GP-reported SDM*** |
| --- | --- | --- | --- | --- |
| ***observer-reported SDM*** | number | 135 | 133 | 131 |
|  | correlation | 1 | 0.123 | 0.418* |
| ***patient -reported SDM*** | number | 133 | 173 | 165 |
|  | correlation | 0.123 | 1 | 0.146 |
| ***GP-reported SDM*** | number | 131 | 165 | 168 |
|  | correlation | 0.418* | 0.146 | 1 |

* =Correlation is significant (p<0.05) at the 0.01 level (2-tailed)
